# Supplementary material for: Automatic detection of the parasite Trypanosoma cruzi in blood smears using a machine learning approach applied to mobile phone images
Source: PeerJ. 2022 May 27;10:e13470. doi: 10.7717/peerj.13470 (PMC9150695; doi:10.7717/peerj.13470)
Supplement: Supplementary file 1 [file peerj-10-13470-s003.odt]

Supplementary information file

Automatic detection of the parasite Trypanosoma cruzi in blood smears using a machine learning approach applied to mobile phone images

Mauro César Cafundó Morais1,2,3,§,†, Diogo M. da Silva1,§, Matheus M. Milagre4, Maykon T. Oliveira4, Thais Pereira5, João S. Silva6, Luciano da F. Costa7, Paola Minoprio2, Roberto Marcondes Cesar Junior8, Ricardo T. Gazzinelli5, Marta de Lana4,9, Helder I. Nakaya1,2,3,10*

1 Department of Clinical and Toxicological Analysis, School of Pharmaceutical Sciences, University of Sao Paulo, São Paulo, 30270-901, Brazil.

2 Scientific Platform Pasteur-University of São Paulo, São Paulo, Brazil.

3 Sociedade Beneficente Israelita Brasileira Albert Einstein (SBIBAE). Instituto Israelita de Ensino e Pesquisa Albert Einstein (IIEPAE), São Paulo, SP, Brazil.

4 Departamento de Análises Clínicas (DEACL), Programa de Pós-graduação em Ciências Farmacêuticas (CiPHARMA), Universidade Federal de Ouro Preto (UFOP), Ouro Preto, MG, 35400-000, Brazil.

5 Laboratório de Imunopatologia, Instituto René Rachou, Fundação Oswaldo Cruz, Belo Horizonte, MG, Brazil. Departamento de Bioquímica e Imunologia, Universidade Federal de Minas Gerais, Belo Horizonte, MG, Brazil.

6 Departamento de Medicina Interna, Faculdade de Medicina de Ribeirão Preto (FMRP), Universidade de São Paulo (USP), Ribeirão Preto, SP, 14049-900, Brazil.

7 São Carlos Institute of Physics, DFCM- IFSC, University of São Paulo, USP.

8 Instituto de Matemática e Estatística (IME), Universidade de São Paulo (USP), São Paulo, SP,  Brazil.

9 Núcleo de Pesquisas em Ciências Biológicas (NUPEB), Universidade Federal de Ouro Preto (UFOP), Ouro Preto, MG, 35400-000, Brazil.

10 Center of Research in Inflammatory Diseases (CRID), University of São Paulo, Ribeirão Preto, SP, Brazil.

§ These authors contributed equally.

† Mauro C. C. Morais

Post-doctoral fellow

University of Sao Paulo Innovation Center (InovaUSP)

University of Sao Paulo, Brazil

Av. Prof. Lucio Martins Rodrigues, 370, Block C / 05508-020

Email address: mauro_morais@usp.br

## Image preprocessing for color conversion to CIEL*a*b* space

We compiled the sequence of calculations used to convert from 24-bit RGB color space to the CIE L*a*b* color space. Initially, the conversion was given by the gamma correction of the R, G and B channel values as follows:

formula

Subsequently, we performed matrix multiplication of the linear values, Clinear ∈ (0,1), to obtain the X Y Z space.

formula

The transformation of matrix [X Y Z]T to CIEL*a*b* was obtained by

formula

where

formula

with formula.

The values of Xn, Yn and Zn were the white reference points. Using Y = 100 normalization, the values of of Xn, Yn and Zn were 95.0475, 100.000 and 108.883, respectively.

Figure S1: Feature selection by principal component analysis (PCA). Proportion of variance of all components in the transformed dataset. The 16 first principal components were selected because they represent 95% of the variance of the data.

Table S1: Prediction performance of models on the training and testing sets after feature selection by PCA.

| Set | Feature selection | Model | Metrics (%) |
| --- | --- | --- | --- |
| Sensitivity | Specificity | Precision | Accuracy | F1-score | AUC |
| Train | PCA | SVM | 91.5 | 83.7 | 85.1 | 87.6 | 88.2 | 0.945 |
|  |  | k-NN | 90.6 | 86.2 | 87.0 | 88.4 | 88.8 | 0.957 |
|  |  | RF | 99.8 | 99.5 | 99.5 | 99.7 | 99.7 | 1.0 |
|  |  | Ensemble | 95.0 | 90.0 | 90.7 | 92.5 | 92.8 | 0.989 |
| Test | PCA | SVM | 48.8 | 58.4 | 51.2 | 53.9 | 50.0 | 0.517 |
|  |  | k-NN | 49.8 | 46.6 | 45.4 | 48.1 | 47.5 | 0.487 |
|  |  | RF | 47.9 | 48.3 | 45.3 | 48.1 | 46.5 | 0.475 |
|  |  | Ensemble | 49.8 | 49.6 | 46.9 | 49.7 | 48.3 | 0.495 |

AUC, Area under the curve. SVM, Support vector machines. k-NN, k-nearest neighbors. RF, random forest.
